# Wandering Fibroid in a Post Menopausal Woman From Rural India: A Case Report

**DOI:** 10.7759/cureus.42734

**Published:** 2023-07-31

**Authors:** Anshul Sood, Roohi G Gupta, Gaurav V Mishra, Shreya Khandelwal, Manasa Suryadevara

**Affiliations:** 1 Radiodiagnosis, Datta Meghe Institute of Higher Education and Research, Wardha, IND

**Keywords:** calcified fibroid, wandering fibroid, leiomyoma, radiodiagnosis, parasitic fibroid

## Abstract

Fibroids, also known as uterine leiomyomas, are the most common solid benign lesions of the uterus. Fibroids are responsive to hormones and are stimulated by estrogens and commonly grow during pregnancy and involute as menopause progresses. The treatment is mostly conservative. When symptomatic, the treatment requires surgical intervention. We present a case of a 72-year-old post-menopausal female with a large, calcified parasitic fibroid, an extremely rare variant of uterine leiomyoma occurring outside the uterus. The number of cases reported about this pathology is minimal.

## Introduction

Leiomyomas are a group of solid benign lesions of the uterus which are primarily responsive to estrogen. Hence, they are mostly seen in menstruating women and tend to aggravate in size during pregnancy and reduce after menopause [1]. Parasitic fibroids are extrauterine leiomyomas and present as peritoneal benign smooth muscle masses separate from the uterus [2]. According to the FIGO classification of fibroids, the parasitic fibroid is grouped under type 8. Broad ligament fibroids are also sometimes classified under parasitic fibroids [3].

## Case presentation

**Figure 1:**
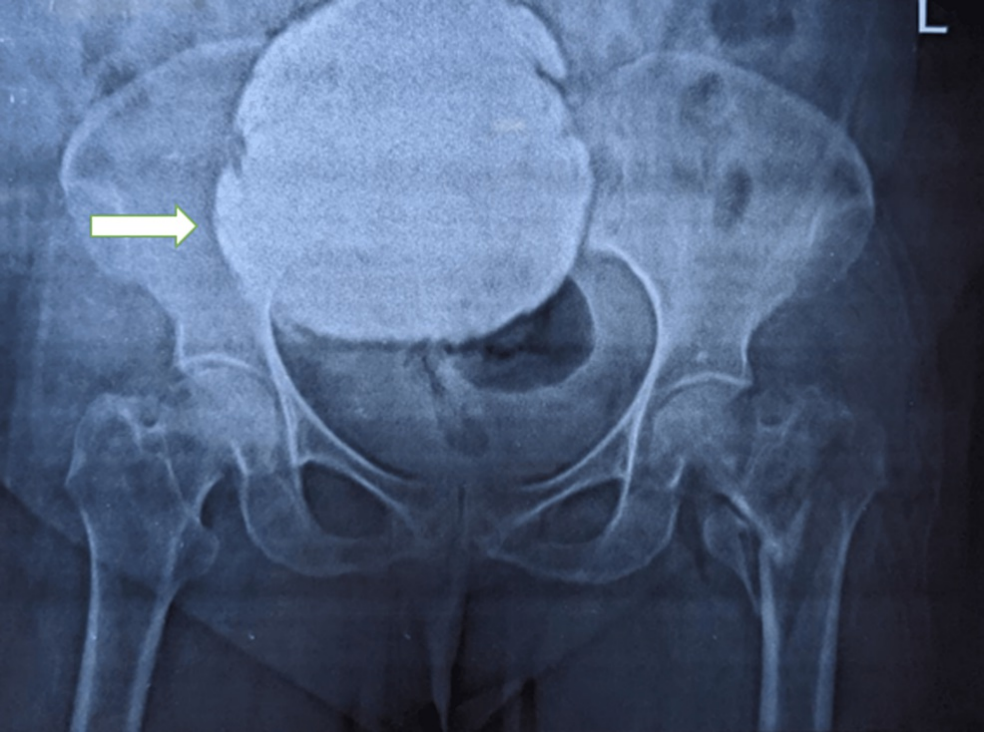
Comminuted displaced fracture of the neck of the left femur with incidental finding of a well-defined radio-opaque mass in the pelvis.

A 72-year-old post-menopausal female came to the orthopedics outpatient department with a history of slip and fall from the stairs, following which the patient could not get up and bear any weight on the left lower limb. Pain and swelling started immediately after the accident, which was sudden in onset and non-progressive in nature. The pain was severe in intensity, aggravated by movement, and partially relieved by rest. The patient was then advised to have an X-ray pelvis, which revealed a comminuted displaced fracture of the neck of the left femur. Incidentally, a well-defined radio-opaque mass lesion was found in the pelvis, separate from the urinary bladder, as shown in Figure 1.

Two days later, the patient complained of pain in the abdomen with a palpable mass in the left lumbar region, freely mobile and not attached to the skin. There was no history of fever, weight loss, or change in bowel and bladder habits. She was para three, live three, and all her children were from normal vaginal delivery. She had no history of any previous surgery and no family history of breast or gynecological malignancy.

**Figure 2:**
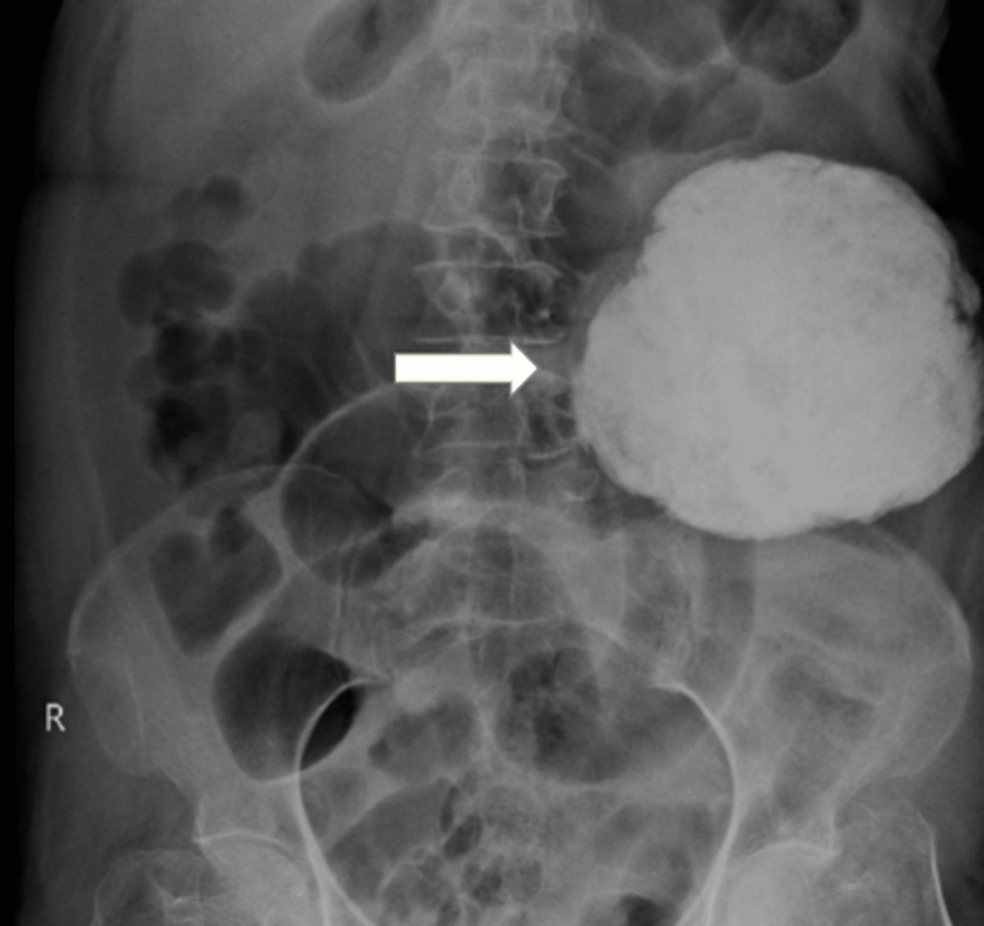
A well-defined radio-opaque mass in the left lumbar and hypogastric region, extending from the splenic flexure to the upper border of the left iliac crest.

The patient was advised to have an X-ray abdomen which revealed a well-defined radio-opaque mass in the left lumbar and hypogastric region, extending from the splenic flexure to the upper border of the left iliac crest, as shown in Figure 2.

**Figure 3:**
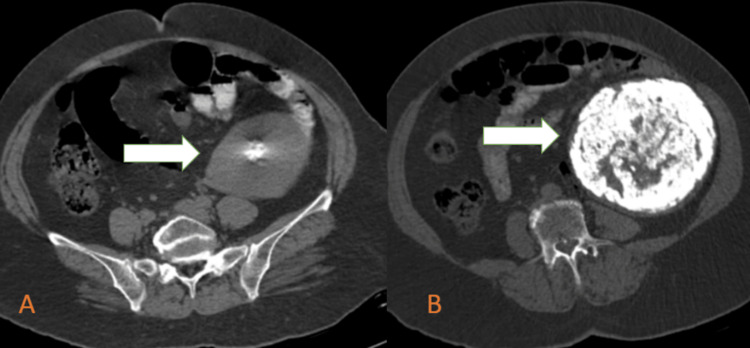
A well-defined, heavily calcified, predominantly hyperdense lesion showing a whorled appearance causing a mass effect in the form of displacement of the left psoas muscle medially.

Sonography of the abdomen and pelvis revealed the presence of a large mass lesion in the left lumbar region extending from the spleen above to the left inguinal ligament below. It was abutting the spleen parenchyma and the left kidney. The lesion was obstructing the ultrasonic waves from passing through, and no organ could be visible posterior to the lesion, suggesting it to be a highly calcified lesion. Further, contrast-enhanced computed tomography (CECT) was advised to get a detailed description of the lesion. CECT revealed a well-defined, heavily calcified, predominantly hyperdense mass lesion showing a whorled appearance of size approximately 12.5 x 12 x 11.5 cm, Hounsfield unit ranging from +630 to +1050, in the left lumbar region inferior to the kidney at the level of L2 to L5 vertebra, not seen separately from the left adnexa, uterus, and the ovaries. Peripherally enhancing soft tissues were noted in continuity with the pelvic soft tissues. A diagnosis of wandering fibroid was made on the basis of imaging. The lesion is causing a mass effect by displacing the left psoas muscle and bowel loops medially. The fat planes, adjacent muscles, and abdominal organs appear normal, as shown in Figure 3.

**Figure 4:**
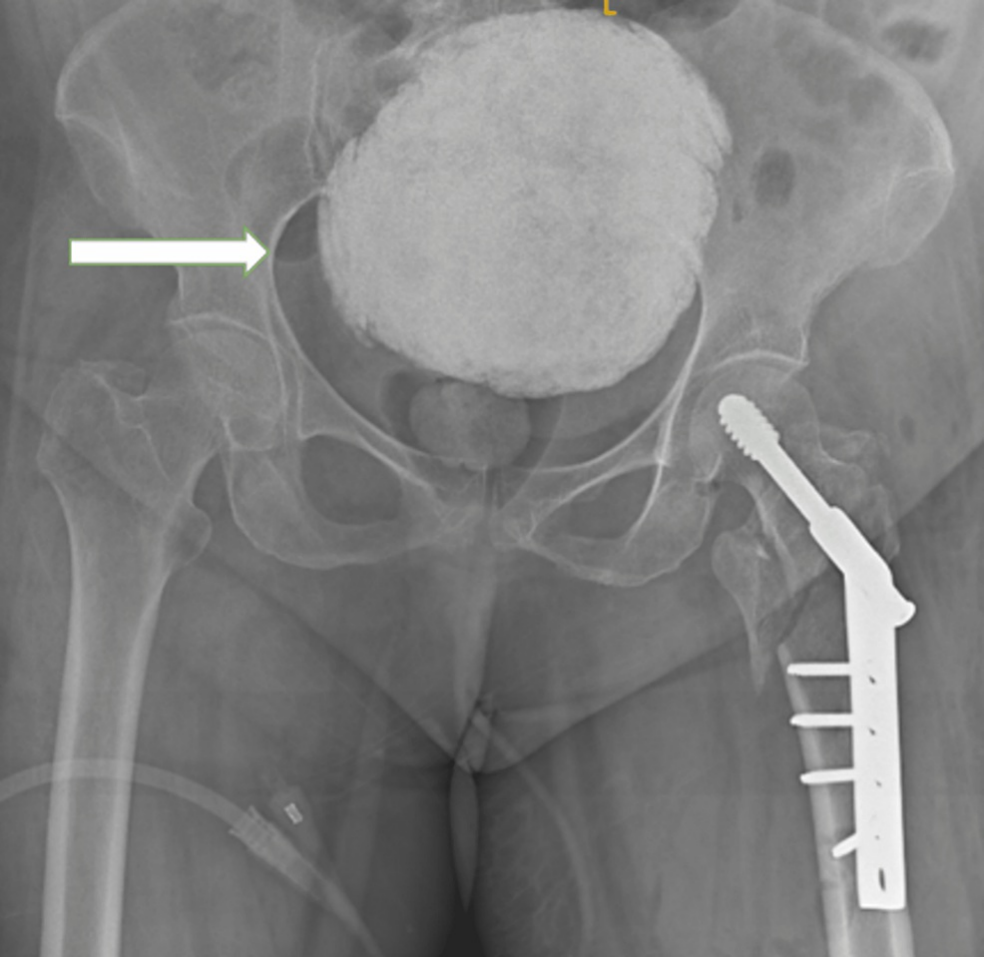
Surgical implant in the neck of the femur with a well-defined radio-opaque lesion in the pelvis.

The patient underwent surgical intervention for a comminuted displaced fracture of the neck of the left femur, after which an X-ray was performed, which shows the same radio-opaque mass in the pelvis and surgical implant in the region of the neck of the left femur as shown in Figure 4.

The patient was advised to follow up for the post-operative implant. An opinion was taken from the surgical team for the fibroid, and they advised conservative management for the same.

## Discussion

Leiomyomas or fibroids are the uterus's most common benign neoplasm, originating from myometrial. These are most commonly composed of smooth muscle cells with variable amounts of fibrous connective tissue. They can be within the uterus and termed as intra-uterine or outside the uterus and termed as extrauterine. Intra-uterine can be classified into intramural, i.e., centered within the myometrium; subserosal, i.e., projecting outwards from the uterus; and submucosal, i.e., projecting into the uterine cavity. Extrauterine leiomyomas are classified into broad ligament leiomyoma, parasitic leiomyoma, and cervical leiomyoma [1]. When multiple poorly defined, confluent leiomyomatous nodules cause almost complete replacement of the myometrium and result in a symmetrically enlarged uterus, it is called diffuse uterine leiomyomatosis [1].

Parasitic leiomyomas are extremely rare, and very few such cases have been published about them. Parasitic leiomyoma was described as a fibroid that has, for some reason, become entirely or partially detached from the uterus and receives its main blood supply from the extrauterine source [4]. Earlier, these were thought to be pedunculated subserosal fibroid which later gets detached from the uterus and gets attached to the non-uterine tissues. Hence, often termed a migrating or wandering leiomyoma. Later, the iatrogenic theory was proposed, which suggested the origin of the parasitic fibroid to be from the seeding of the leftover tissues of the fibroids, which were thought to be produced by previous laparoscopic myomectomy, most commonly by morcellation [4-9].

The symptoms related to parasitic fibroids are usually vague, and the patient may present with pain in the abdomen, heaviness, or a palpable lump in the abdomen which can be migratory. Treatment is usually surgical, which can be either laparoscopic or open surgery.

This pathology might be challenging to differentiate from ovarian or retroperitoneal tumors, as in our case.

One of the rarest and severe complications of this entity is the rupture of the uterus and very few cases have been reported about this entity [10,11]. An abdominal CT scan is extremely useful in diagnosing such complications [12].

## Conclusions

Parasitic fibroids are an extremely rare group of leiomyomas under type 8 of the FIGO classification of uterine leiomyomas. This type of leiomyoma gets its blood supply from the extrauterine source. They increase in size similar to the other leiomyomas, i.e., by estrogens, and commonly grow during pregnancy and start to involute with menopause. The primary treatment of parasitic fibroid is surgical intervention by either laparoscopic or open surgery. Minimal cases have been published about this pathology, and it is essential to differentiate it from other differentials like ovarian or retroperitoneal tumors.
